# The Adipose Tissue-Derived Secretome (ADS) in Obesity Uniquely Induces L-Type Amino Acid Transporter 1 (LAT1) and mTOR Signaling in Estrogen-Receptor-Positive Breast Cancer Cells

**DOI:** 10.3390/ijms22136706

**Published:** 2021-06-23

**Authors:** Chelsea Thompson, M Motiur Rahman, Soudamani Singh, Subha Arthur, Cecilia Sierra-Bakhshi, Rebecca Russell, Krista Denning, Uma Sundaram, Travis Salisbury

**Affiliations:** 1 Department of Biomedical Sciences and Appalachian Center for Cellular Transport in Obesity Related Disorders, Joan C. Edwards School of Medicine, Marshall University, 1 John Marshall Drive, Huntington, WV 25755, USA; thompsonch@marshall.edu (C.T.); sierra3@live.marshall.edu (C.S.-B.); 2 Department of Clinical and Translational Sciences and Appalachian Clinical and Translational Science Institute and Appalachian Center for Cellular Transport in Obesity Related Disorders, Joan C. Edwards School of Medicine, Marshall University, 1600 Medical Center Drive, Huntington, WV 25701, USA; rahmanmd@marshall.edu (M.M.R.); singhs@marshall.edu (S.S.); arthursu@marshall.edu (S.A.); sundaramu@marshall.edu (U.S.); 3 Cabell Huntington Hospital Laboratory, Department of Pathology and Appalachian Center for Cellular Transport in Obesity Related Disorders, Joan C. Edwards School of Medicine, Marshall University, Huntington, WV 25701, USA; Rebecca.Russell@chhi.org (R.R.); haught5@marshall.edu (K.D.)

**Keywords:** breast cancer, obesity, LAT1, leucine, mTOR

## Abstract

Obesity increases the risk of postmenopausal breast cancer (BC). This risk is mediated by obesity-induced changes in the adipose-derived secretome (ADS). The pathogenesis of BC in obesity is stimulated by mTOR hyperactivity. In obesity, leucine might support mTOR hyperactivity. Leucine uptake by BC cells is through L-Type Amino Acid Transporter 1 (LAT1). Our objective was to link obesity-ADS induction of LAT1 to the induction of mTOR signaling. Lean- and obese-ADS were obtained from lean and obese mice, respectively. Breast ADS was obtained from BC patients. Estrogen-receptor-positive BC cells were stimulated with ADS. LAT1 activity was determined by uptake of ^3^H-leucine. The LAT1/CD98 complex, and mTOR signaling were assayed by Western blot. The LAT1 antagonists, BCH and JPH203, were used to inhibit LAT1. Cell migration and invasion were measured by Transwell assays. The results showed obese-ADS-induced LAT1 activity by increasing transporter affinity for leucine. Consistent with this mechanism, LAT1 and CD98 expression were unchanged. Induction of mTOR by obese-ADS was inhibited by LAT1 antagonists. Breast ADS from patients with BMIs > 30 stimulated BC cell migration and invasiveness. Collectively, our findings show that obese-ADS induction of LAT1 supports mTOR hyperactivity in luminal BC cells.

## 1. Introduction

In the United States, 70% of adults are overweight and more than one-third (~40%) are obese [1,2]. Prior reports show that obesity is an important risk factor for postmenopausal breast cancer (BC) [3,4,5]. The effect of obesity on BC is selective for estrogen-receptor (ER)-positive BC [3,4,5]. Obesity is significantly associated with larger tumor size, positive lymph nodes, metastatic disease, higher risk of BC recurrence and significantly higher rates of BC mortality [3,4,5,6,7]. When considering there are approximately 70 million postmenopausal women in the United States [8] and that potentially 70% of these women are overweight to obese, it is possible that obesity places 28 million women at a greater risk for invasive BC in the U.S. 

Adipose tissue (AT) plays a key role in linking obesity with increased risk for BC. Obesity leads to pathological changes in AT, which in turn lead to a cancer-promoting AT-derived secretome (ADS) [9,10,11,12]. The obese-ADS consists of proinflammatory cytokines, altered levels of adipokines (increased leptin and reduced adiponectin), lipids, and AT-derived extracellular vesicles that act on BC cells to induce signaling that promotes proliferation, cell migration and cancer cell invasiveness [10,11,12]. 

Mechanistic target of rapamycin (mTOR) is an atypical serine/threonine kinase that is part of two large protein complexes known as mTOR complex 1 (mTORC1) and mTOR complex 2 (mTORC2) [13,14,15,16,17]. mTORC1 is the major intracellular pathway that responds to nutrients such as amino acids [13,14,17,18,19,20,21,22]. The activation of mTORC1 in turn regulates protein synthesis, lipid synthesis, cellular metabolism, autophagy, cell growth and proliferation [13,14,17,23]. Nutrient overload in obesity promotes mTORC1 hyperactivity in adipose tissue, muscle and in some cancers, such as postmenopausal breast cancer and colon cancer [10,13,14,17,24,25,26]. The concentration of leucine is significantly (*p* < 0.0001) higher (by 14%) in obese subjects compared with lean subjects [27]. Given that leucine can stimulate and sustain mTOR activation [28], increased levels of this amino acid could in turn contribute to sustained mTOR hyperactivity in obesity. This hypothesis, however, is contingent on robust uptake of leucine by cancer cells in obesity. Regarding BC, this link would be mediated through the leucine transporter, L-Type Amino Acid Transporter 1 (LAT1), which we and others have shown is the primary mechanism by which BC cells absorb extracellular leucine [29,30,31,32]. 

The primary uptake of large branched neutral amino acids, such as leucine, by cells is through system-L amino acid transporters [32,33,34]. Among system-L, LAT1 is the primary leucine transporter in luminal ER-positive BC cells [29,30,31]. LAT1 transports amino acids as a complex covalently bound to 4F2 Heavy-Chain Antigen (also called CD98 and official gene name: SLC3A2) [33,34,35]. CD98 promotes LAT1 protein stability, mediates the translocation of LAT1 to the cell membrane and is required for LAT1 transport function [31,33,34]. To date, most reports have focused on transcriptional regulation of LAT1 [36]. We have shown that, in BC cells, LAT1 transcription is stimulated by the aryl hydrocarbon receptor (AHR) [29]. However, the regulation of LAT1 affinity for leucine has not been reported to date. Further, there are no reports showing that obesity regulates the activity of LAT1. 

Based on the premise detailed above, in this study we investigated obese-ADS regulation of LAT1 in luminal ER-positive BC cells. We show that compared with lean-ADS, obese-ADS induced LAT1 activity in BC cells by increasing LAT1 affinity for leucine at physiologically relevant leucine concentrations. We show that increases in leucine affinity are linked with greater sensitivity of the mTOR pathway to obese-ADS compared with lean-ADS. These functional and molecular changes in LAT1 and mTOR were correlated with enhanced luminal BC cell migration and invasiveness in response to obese-ADS. To our knowledge, these findings are the first to link obesity with the regulation of LAT1 in luminal BC cells and provide a new mechanism that mediates mTOR hyperactivity in obesity in BC.

## 2. Results

### 2.1. Obese-ADS Stimulates LAT1 Activity

**Figure 1:**
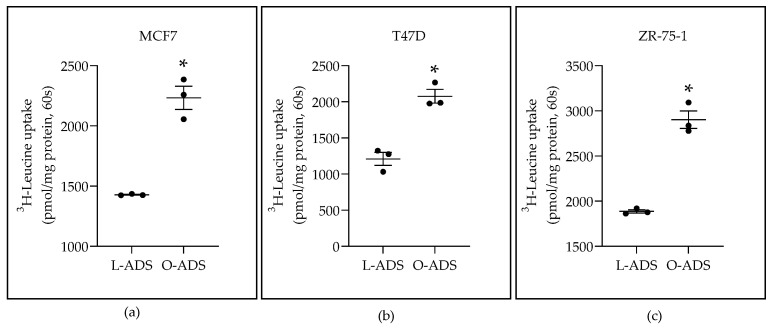
The effect of lean (L) and obese (O) AT-derived secretome on LAT1 activity in: (**a**) MCF7 cells; (**b**) T47D cells; (**c**) ZR-75-1 cells. Visceral AT was isolated from lean (L) and obese (O) mice. Pieces of AT were cultured for 24 h. After culture, the media were collected and centrifuged to remove tissue debris. The cleared media, which is the AT-derived secretome (ADS), were diluted 1:10 in media with 0.1% FBS and placed on ER-positive BC cells for 24 h (two times at 12 h intervals) for ^3^H-Leucine uptake analysis. Significant induction by O-ADS compared with L-ADS, based on Student’s *t*-test analysis, is indicated by * *p* < 0.05 (*n* = 3). The data are shown as the mean ± SEM (error bars).

We sought to define regulation of LAT1 in breast cancer (BC) in obesity. To define an adipose tissue (AT) effect, AT-derived secretome (ADS) was placed on human luminal ER-expressing BC cells. Our premise is that regulation of LAT1 is the sum of positive and negative signals from AT, instead of the effect of a single factor. In our studies, lean-ADS (L-ADS) was obtained from visceral fat from lean mice. Obese-ADS (O-ADS) was obtained from visceral fat from high-fat-diet-fed obese mice. Luminal ER-positive BC cells were replenished with ADS in media every 12 h for 24 h. The results showed that LAT1 activity, defined as BCH-sensitive uptake of ^3^H-Leucine, was significantly induced in MCF7, T47D, and ZR-75-1 BC cells by O-ADS compared with L-ADS (Figure 1). 

**Figure 2:**
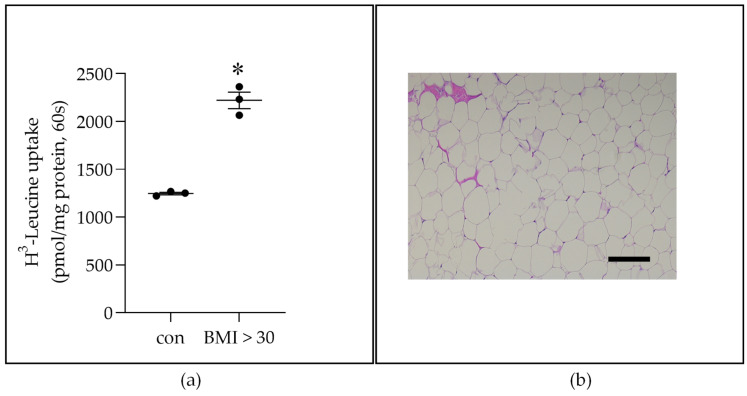
The effect of peritumor (PT) breast AT-derived secretome (PT-ADS) from women with BMIs > 30 on LAT1 activity in ER-positive ZR-75-1 cells. (**a**) Breast AT was obtained from women with breast cancer. Pieces of breast AT were cultured for 24 h. After culture, the media were collected and centrifuged to remove tissue debris. The cleared media, which is PT-ADS, were diluted 1:10 in media with 0.1% FBS and placed on ER-positive ZR-75-1 cells for 24 h (two times at 12 h intervals) for ^3^H-Leucine uptake analysis. The asterisk (*, *p* < 0.05) indicates a significant, based on Student’s *t*-test analysis, increase in ^3^H-Leucine uptake in response to PT-ADS from women with BMI’s > 30 compared with base-level uptake by control (con) ZR-75-1 cells. The data are shown as the mean ± SEM (*n* = 3). (**b**) H&E stain showing adipocytes in breast AT used to generate PT-ADS (magnification 100×). Scale bar is 100 µm.

Considering paracrine mechanisms, we tested the effect of breast peritumor (PT) ADS (PT-ADS). Breast PT-ADS was obtained from breast AT donated by BC patients (BMI > 30) (*n* = 3). The BC patients were treatment-naïve. The results showed that, relative to the no-ADS control (con), PT-ADS induced LAT1 activity in ZR-75-1 BC cells (Figure 2a). The percentage of adipocytes in breast adipose used to generate PT-ADS was nearly 100% (Figure 2b). Collectively, these data show that obese-ADS induces LAT1 activity, as defined as BCH-sensitive uptake of ^3^H-Leucine, in luminal ER-positive BC cells. 

### 2.2. Obese-ADS Increases LAT1 Affinity for Leucine

**Figure 3:**
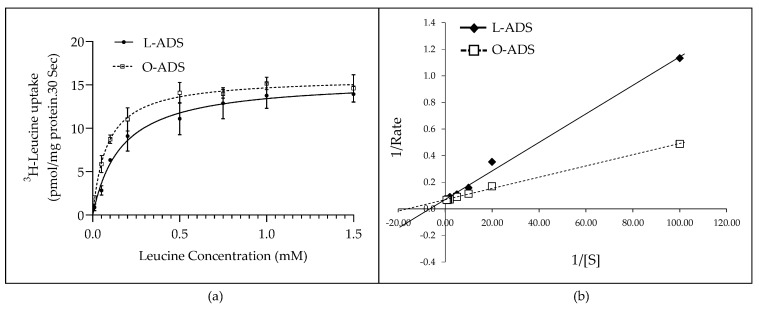
Kinetic study of ^3^H-leucine uptake by MCF7 breast cancer (BC) cells. The method by which mouse AT-derived secretome (ADS) was isolated and placed on ER-positive MCF7 cells for the kinetic study is indicated in the legend of Figure 1. (**a**) As the concentration of ^3^H-Leucine was increased, its uptake was stimulated and subsequently became saturated in response to lean-ADS (L-ADS) and obese-ADS (O-ADS) (*n* = 3). (**b**) Analysis of the data in panel (**a**) with Lineweaver Burk plot yielded kinetic parameters. The maximal uptake of ^3^H-Leucine was not different in response to O-ADS. However, the affinity for ^3^H-Leucine was increased in response to O-ADS compared with L-ADS.

**Figure 4:**
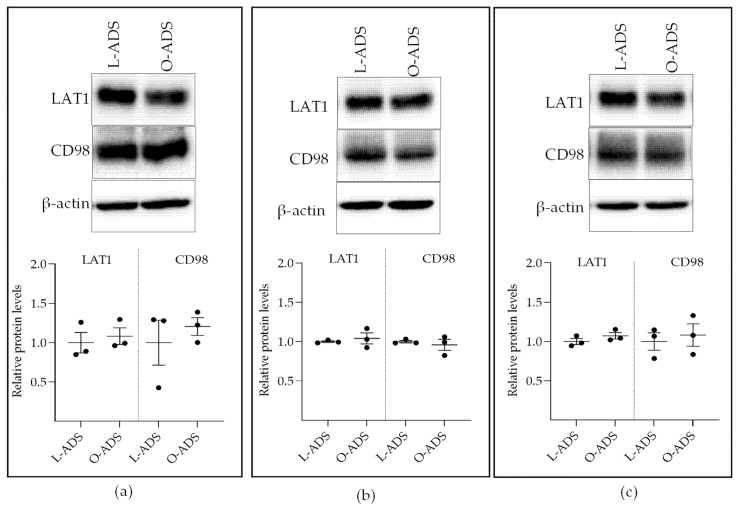
The effect of lean (L)- and obese (O)-ADS on LAT1 and CD98 protein expression. The method by which mouse AT-derived secretome (ADS) was isolated and placed on ER-positive BC cells for Western blot analysis is indicated in the legend of Figure 1. Relative protein levels of LAT1 and CD98 were calculated as the densitometric signal for LAT1 and CD98 divided by the densitometric signal for β-actin loading control. Statistical analysis, based on Student’s *t*-test analysis, showed that the levels of LAT1 and CD98 were not changed in response to O-ADS compared with L-ADS in: (**a**) MCF7; (**b**) T47D; and (**c**) ZR-75-1 cells. The data are shown as the mean ± SEM (error bars) for three experiments (*n* = 3).

Next, experiments were conducted to determine the mechanism by which O-ADS increases LAT1 activity. To this end, kinetic studies were conducted in ER-positive MCF7 BC cells. The results show that O-ADS stimulated LAT1 affinity (1/Km) for leucine without changing the maximal rate (*Vmax*) of leucine uptake (Figure 3a). Lineweaver Burk Plot analysis confirmed increases in LAT1 affinity (1/Km) for leucine without changing the maximal rate (*Vmax*) of leucine uptake in response to O-ADS compared with L-ADS (Figure 3b). The specific kinetic parameters are shown in Table 1. Consistent with this mechanism, O-ADS did not change the levels of LAT1, or its chaperon protein, CD98, protein in ER-positive BC cells (Figure 4). Collectively, these data show that O-ADS increases LAT1 activity by stimulating LAT1 affinity for leucine and not by increasing the number of LAT1 transporters.

### 2.3. LAT1 Mediates Obese-ADS Induction of mTOR

**Figure 5:**
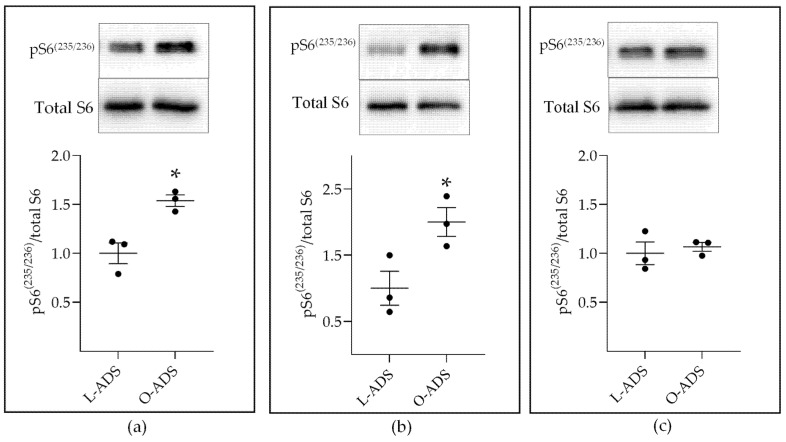
The effect of lean (L)- and obese (O)-ADS on mTOR signaling in: (**a**) MCF7; (**b**) T47D; and (**c**) ZR-75-1 cells. The method by which mouse AT-derived secretome (ADS) was isolated and placed on ER-positive BC cells for Western blot analysis is indicated in the legend of Figure 1. * Indicates a significant (*p* < 0.05) increase in phosphorylated ribosomal S6 protein on serine 235/236 (pS6^235/236^) in response to O-ADS compared with L-ADS, as analyzed by Student’s *t*-test (*n* = 3). All data represent mean ± S.E.

The kinetics study showed that leucine concentrations showing the greatest fold increase in uptake in response to O-ADS were between 10 and 200 µM (Figure 3). To study the regulation of mTOR without the confounding effects of high leucine concentration (800 µM) in media [37], cells were replenished with ADS in media with a lower concentration (45 µM) of leucine every 12 h for 24 h. The results showed that O-ADS stimulated increases in mTOR signaling as measured by increased phospho-S6^235/236^ over total S6 Ribosomal protein (S6) in MCF7 and T47D BC cells (Figure 5a,b). The basal activity of the mTOR pathway in ZR-75-1 was high and not increased by O-ADS (Figure 5c). 

**Figure 6:**
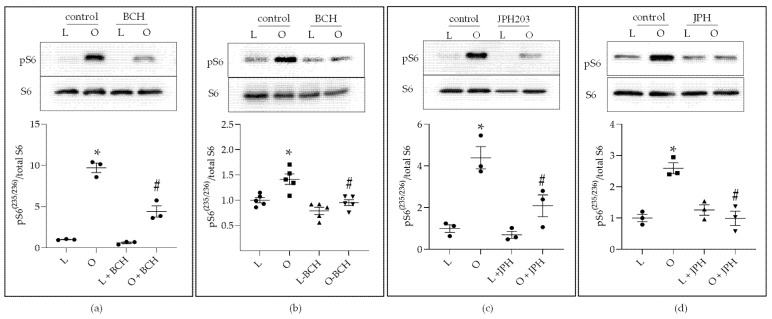
The effect of BCH and JPH203 on mTOR signaling: ER-positive BC cells were treated with AT-derived secretome (ADS) from lean and obese mice as described in the legend of Figure 1 with vehicle, BCH (10 mM) or JPH203 (JPH) (15 µM). Results in MCF7 cells are shown in (**a**,**c**). Results in T47D cells are shown in (**b**,**d**). * Indicates a significant (*p* < 0.05) induction of phospho-S6^(235/236)^ by obese-ADS (O) compared with lean-ADS (L). ^#^ Indicates a significant (*p* < 0.05) decrease in phospho-S6^(235/236)^ compared with obese-ADS (O). (**a**,**c**,**d**) experiments were repeated three times (*n* = 3). (**b**) experiments were repeated five times (*n* = 5). Significant differences were determined by two-way ANOVA, followed by Tukey’s multiple comparisons test. Data shown represent the mean ± SE.

We hypothesized that LAT1 mediated increases in mTOR signaling. To investigate this hypothesis, we inhibited LAT1 activity with two LAT1 antagonists, BCH [38] and JPH203 [39]. Relative to L-ADS (L), O-ADS (O) induced mTOR signaling in vehicle-treated (control) MCF7 and T47D cells (Figure 6). This induction was inhibited by BCH (10 mM) (Figure 6a,b) and JPH203 (15 µM) (Figure 6c,d). These data suggest that LAT1 mediates increases in mTOR signaling in response to O-ADS.

### 2.4. Obese-ADS Stimulates BC Cell Migration and Invasiveness

**Figure 7:**
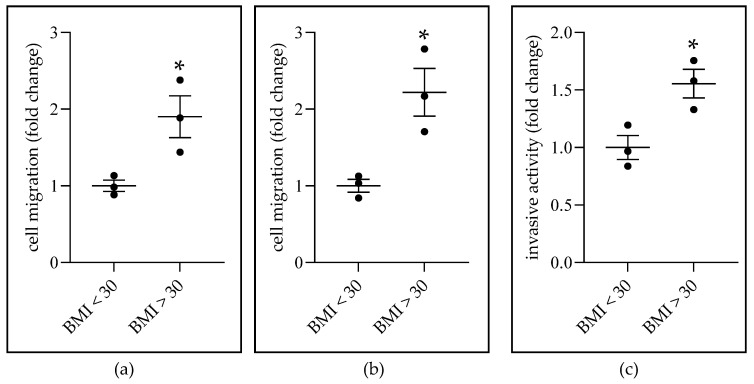
The effect of peritumor (PT) breast AT-derived secretome (PT-ADS) from women with BC on ER-positive BC migration and invasion. (**a**,**b**), MCF7 cells were treated with PT-ADS diluted 1:10 in media with 0.1% FBS for (**a**) 12 h or (**b**) 24 h for cell migration analysis. (**c**) T47D invasion toward PT-ADS diluted 1:10 in media with 20% FBS for 4 days (treated one time). * Indicates a significant (*p* < 0.05) increase, based on Student’s *t*-test analysis, in cell migration and invasion in response to PT-ADS from women with BMI > 30 compared with PT-ADS from women with BMI < 30. The data shown represent the mean ± SEM (*n* = 3).

Finally, we questioned the effect of ADS on the migration and invasiveness of ER-positive BC cells. Experiments were carried out with breast PT-ADS. Breast PT-ADS was obtained from treatment-naïve BC patients. The results showed that PT-ADS obtained from patients with BMIs > 30 induced greater MCF7 cell migration (Figure 7a,b) and T47D cell invasiveness (Figure 7c) than PT-ADS obtained from patients with BMIs < 30. These results show that BMI and PT-ADS are functionally linked with inducing migratory and invasive activity of luminal BC cells. 

## 3. Discussion

This study is the first to show that ADS increases LAT1 activity. The induction of LAT1 was linked to obesity because increases in LAT1 activity were significantly greater in response to O-ADS compared with L-ADS (Figure 1, Figure 2 and Figure 3 and Table 1). We show that LAT1 is induced in ER-expressing BC cells, which is the BC subtype that is linked with increased risk in obesity (Figure 1, Figure 2 and Figure 3 and Table 1) [3,4,5]. Using molecular and functional techniques, we show that the increase in LAT1 activity is due to an increase in leucine affinity and not due to increased number of LAT1/CD98 transporters (Figure 1, Figure 2, Figure 3 and Figure 4 and Table 1). We show that the induction of mTOR is significantly stronger in response to O-ADS compared to L-ADS (Figure 5 and Figure 6). The induction of mTOR was reduced by LAT1 antagonists, BCH and JPH203 (Figure 6). Therefore, O-ADS induction of mTOR in luminal BC cells is in part mediated by LAT1. These novel findings suggest that inhibiting mTOR by targeting LAT1 could be particularly relevant for ER-positive BC in obesity. 

The kinetic study showed that O-ADS stimulates LAT1-mediated uptake of physiologically relevant concentrations of leucine (Figure 3). The greatest fold increase in leucine uptake by LAT1 in response to O- compared to L-ADS occurred at leucine concentrations between 10 and 200 µM (Figure 3), which overlaps the range of leucine concentrations in human plasma (66–170 µM) [27,37]. These data suggest that, in obesity, breast tumors have a higher affinity for plasma leucine than breast tumors of lean subjects.

Our mTOR results are consistent with and extend the mTOR findings of prior reports. For instance, plasma from obese subjects is a stronger inducer of mTOR signaling in MCF7 cells than plasma from lean subjects [40]. Mammary tumors in obese female mice showed higher mTOR signaling than mammary tumors in lean mice [41]. The mTOR inhibitor, everolimus, reduced mammary tumor size in obese mice [41]. In human subjects, body fatness is linked with mTOR pathway activation in BC [42]. Our findings extend the findings of these prior studies by providing strong evidence that intact obese visceral AT secretes factors that act on ER-positive BC cells to stimulate increases in mTOR signaling compared with lean visceral AT (Figure 5 and Figure 6). 

A recent report shows that high expression of LAT1 in breast tumors is associated with poor patient response to tamoxifen [31]. Consistent with therapy resistance, overexpression of LAT1 induced tamoxifen resistance in MCF7 cells [31]. Further, BCH restored tamoxifen sensitivity to tamoxifen-resistant MCF7 cells [31]. Clinical findings show that LAT1 is a biomarker for tamoxifen resistance [43]. Our data show that obese-ADS stimulates LAT1 activity in three ER-positive BC cell lines, including MCF7 (Figure 1). Based on these prior reports and our findings, obesity might affect tamoxifen response due to changes in LAT1 affinity for leucine in breast tumors. 

JPH203 is a selective LAT1 antagonist [39]. It has anticancer activity, reduces mTOR signaling, inhibits the proliferation of cancer cells in cell culture and suppresses the growth of various tumors (including luminal ER-positive breast tumors) in murine cancer models [31,36,44,45]. JPH203 has been evaluated in a human phase I clinical trial and it was shown to be well tolerated [46]. JPH203 activity is dependent on the concentration of leucine in cell culture media, which suggests that it acts as a competitive antagonist of LAT1 [47]. Consequently, it is possible that JPH203 efficacy in humans could be affected by fluctuations in the concentration of leucine in human plasma. Based on our data, it is also possible that JPH203 efficacy could be affected by changes in LAT1 affinity for leucine in obesity. 

Epidemiological studies link obesity with greater risk for metastatic BC [3,7]. It is possible that this risk is mediated by breast AT. For instance, murine mammary adipose stem cells are strong inducers of mammary cancer invasiveness, if the adipocytes are isolated from obese mice [48]. Breast adipose stem cells from noncancerous breast tissue obtained from women stimulates MCF7 migration and invasiveness, if the adipocytes are isolated from women that have a BMI > 30 [48]. We show a similar link between BMI and breast AT stimulation of luminal BC cell migration and invasiveness (Figure 7). Interestingly, the previous report established the link using breast adipose cells from breast tissue that did not have cancer [48]. We show the same link using intact breast AT donated by women with BC (Figure 7). Together, these data show that BMI affects the cell-invasive-inducing activity of breast AT irrespective of BC. 

In this study, there was an increase in leucine affinity rather than a change in the maximum velocity of leucine uptake by LAT1 in MCF7 cells in response to O-ADS (Figure 3). Consistent with this finding was LAT1 and CD98 protein expression, which did not change (Figure 4). It is possible that O-ADS increased LAT1 affinity for leucine by regulating the phosphorylation of LAT1. Cytosol-facing residues on LAT1 that could potentially be phosphorylated are serine 31, serine 33, and threonine 45, which are located on the N-tail of the protein [49]. However, to our knowledge, the regulation of LAT1 phosphorylation has not been reported. Therefore, future studies will be required to establish the regulation and functional significance of putative phosphorylation sites in LAT1. 

## 4. Materials and Methods

### 4.1. Cell Culture and Reagents

Luminal estrogen-receptor (ER)-positive breast cancer (BC) cell lines (MCF7, T47D, ZR-75-1) were acquired from the American Type Culture Collection (Manassas, VA, USA) and cultured at 37 °C with 5% CO_2_. MCF7 was cultured in DMEM/F12 with 10% fetal bovine serum (FBS) and Penicillin-Streptomycin (P/S). T47D and ZR-75-1 were cultured in RPMI-1640 with 10% FBS and P/S. Media, P/S and FBS were purchased from Thermo Fisher Scientific (Waltham, MA, USA). 2-Amino-2-norbornanecarboxylic acid (BCH), JPH203 (KYT-0353) and Dimethyl sulfoxide (DMSO) were acquired from Sigma-Aldrich (St. Louis, MO, USA). Stock BCH (100 mM) was prepared in media. Stock JPH-203 (1 mg/mL; 2.1 mM) was prepared in DMSO. Antibodies were acquired from Cell Signaling Technology (Danvers, MA, USA): anti-LAT1 (rabbit), anti-CD98 (rabbit), anti-S6 Ribosomal Protein (rabbit), β-Actin (rabbit), anti-Phospho-S6 Ribosomal Protein (Ser^235/236^). 

### 4.2. Mouse Obesity Model

Female C57BL/6J mice were obtained from The Jackson Laboratories (Bar Harbor, ME, USA). Mice were maintained in a 12 h light/12 h dark cycle. Mice were fed with a low-fat diet (10% calories from fat; TestDiet 58Y2) or high-fat diet (60% calories from fat; TestDiet 58Y1) from 5 to 20 weeks of age. TestDiets were purchased from Research Diets, Inc. (New Brunswick, NJ, USA). Sixteen weeks post diet, mice were euthanized, and visceral white adipose tissue (AT) from lean and obese mice was collected for AT culture. Animals were handled and euthanized according to Marshall University’s Institutional Animal Care and Use Committee’s ethics and regulation guidelines as accredited by the Association for Assessment and Accreditation of Laboratory Animal Care. Ethical code number: 665. Date of approval: 14.01.2020. 

### 4.3. Human Samples 

De-identified peritumor (PT) breast AT samples were obtained from the biorepository bank at the Edwards Comprehensive Cancer Center at Cabell Huntington Hospital, Huntington, WV. De-identified samples were obtained with patient consent. Breast AT samples (~100 mg) were obtained from women with BC. The patients were treatment-naïve. AT samples were placed into capped vials and transported on ice to the laboratory as soon as possible, within 60 min of surgery. Upon arrival at the laboratory, tissue samples were immediately processed for AT culture.

### 4.4. AT-Derived Secretome (ADS) System

Isolated AT was rinsed and then cut into equal-sized pieces (~10 mg (2–4 mm^3^) per piece). Clean AT pieces were cultured (~100 mg/mL for mouse tissue and ~20 mg/mL for human tissue) at 37 °C in 5% CO_2_. After 24 h of AT culture, the AT-conditioned media (reflects ADS) were collected and centrifuged at 10,000 rpm (at 4 °C) for 10 min to pellet tissue debris. Rinsing, cutting, and culturing were done in phenol red-free, serum-free DMEM/F12. ADS was diluted 1:10 in media with 0.1% FBS before being placed on BC cells. Diluting ADS (1:10) dilutes harmful metabolites that might leach out from AT in culture. 

### 4.5. Western Blotting Studies

In LAT1, CD89 studies, at 1 day post confluence, cells in 6-well tissue culture plates were cultured in DMEM/F12 (MCF7) or RPMI-1640 (T47D and ZR-75-1) with 0.1% FBS for 1 day. Cells were then replenished with 10% ADS in DMEM/F12 (MCF7) or RPMI-1640 (T47D and ZR-75-1) with 0.1% FBS every 12 h for 24 h. For mTOR signaling studies, cells were replenished with 10% ADS in RPMI-1640 media with leucine at a final concentration of 45 µM every 12 h for 24 h. We customized the amount of leucine in media by diluting ADS in leucine-free RPMI-1640 (MP Biologicals, Solon, OH, USA, # 091629149) supplemented with L-glutamine (MP Biologicals, Solon, OH, USA) (2.0 mM). Cells were rinsed twice with leucine-free media (Sigma-Aldrich, St. Louis, MO, USA # R1780) and cultured for 24 h in leucine low (4 µM) RPMI-1640 prior to ADS treatment. The same was done for the LAT1 antagonist studies, with cells being refreshed with 10% ADS supplemented with BCH (10 mM) or JPH-203 (14 µM). 

Post treatment and PBS wash, cells were scraped in 300 µL of cells lysis buffer (Tris-HCl (pH 7.5), 150 mM NaCl, 1 mM Na2EDTA, 1 mM EGTA, 1% Triton, 2.5 mM sodium pyrophosphate, 1 mM b-glycerophosphate, 1 mM Na3 VO4, 1 µg/mL leupeptin) (Cell Signaling Technology, Danvers, MA, USA, #9803) with halt protease and phosphatase inhibitor cocktail (Thermo Fisher Scientific, Waltham, MA, USA). Cell lysate was transferred to microtubes, sonicated twice for 30 s on ice, and centrifuged at 10,000 rpm to pellet cell debris. Extracts were heat-denatured in Laemmli sample buffer with β-mercaptoethanol. Proteins (~30 µg/well) were separated by SDS/PAGE and transferred to polyvinylidene difluoride membranes. Blots were incubated overnight with primary antibody (LAT1 dilution: 1:1000 and CD98, total S6, phospho-S6 and β-actin dilution, 1:2000)) and secondary antibody (diluted 1:5000) for 3 h or overnight at room temperature. Blots were rinsed with 1X Tris Buffered Saline Tween 20 (0.1%) (TBST) three times (five minutes per wash) after incubation with primary and secondary antibody. Western signals were detected using chemiluminescence (Bio-Rad Laboratories, Hercules, CA, USA). LAT1 and CD98 signals were normalized to β-actin and phospho-S6^235/236^ was normalized to total S6. The ChemiDoc MP Imaging System (image lab 4.0) was used to quantify band density and acquire Western blot images (Bio-Rad Laboratories, Hercules, CA, USA). Automatically detected bands were analyzed for volume intensity, which is the sum of all intensities within the lane boundaries, with background subtraction (image lab 4.0).

### 4.6. Leucine Uptake Study

At 1 day post confluence, cell monolayers in 24-well plates were cultured in DMEM/F12 (MCF7) or RPMI-1640 (T47D, ZR-75-1) with 0.1% FBS for 1 day. Cells were then replenished with 10% ADS in DMEM/F12 (MCF7) or RPMI-1640 (T47D and ZR-75-1) with 0.1% FBS every 12 h for 24 h. At 24 h post ADS treatment, cells were washed twice with Na-HEPES buffer (130 mM NaCl, 5 mM KCl, 1 mM MgSO4, 2 mM CaCl2, 20 mM HEPES; pH 7.4) and incubated with the same for 10 min at room temperature. Uptake was then initiated by incubating the cells for 1 min with Na-free buffer (130 mM TMACl, 5 mM KCl, 1 mM MgSO4, 2 mM CaCl2, 20 mM HEPES; pH 7.4) with 10 μCi of ^3^H-L-Leucine (PerkinElmer; Waltham, MA, USA) and 200 μM L-Leucine (Sigma-Aldrich, St. Louis, MO, USA) medium in the presence or absence of 1 mM BCH, a Na(+)-independent system L specific inhibitor. The reaction was stopped with ice-cold Na-HEPES buffer, after which the cells were washed twice with the same ice-cold buffer. The cells were then lysed in 400 μL of 1 N NaOH, followed by incubation for 20 min at 70 °C. The lysed contents of each well were collected in scintillation vial and mixed with 4 mL Ecoscint A (National Diagnostics; Atlanta, GA, USA). Radioactivity was determined in a Beckman 6500 Beta Scintillation Counter (PerkinElmer; Waltham, MA, USA). Na(+)-independent uptake was calculated by subtracting uptake with and without BCH.

### 4.7. Kinetics Study for Leucine Uptake

MCF7 cells were replenished with 10% ADS in media with 0.1% FBS every 12 h for 24 h. The uptake reaction was started by adding Na-free buffer as mentioned above with 10 μCi of ^3^H-L-Leucine and varying concentrations of L-Leucine (0.01 mM, 0.05 mM, 0.1 mM, 0.2 mM, 0.5 mM, 0.75 mM, 1 mM and 1.5 mM) with or without 1 mM BCH. The uptake was stopped at 30 s with the addition of ice-cold Na-HEPES buffer, and radioactivity was determined as described above. Uptake numbers that were obtained from these experiments were analyzed with GraphPad Prism 8 (GraphPad Software Inc., San Diego, CA, USA) for Michaelis–Menten kinetics using a nonlinear regression data analysis to derive kinetic parameters *V_max_* and *K_m_*.

### 4.8. Migration Assay

MCF7 cell migration was determined using the xCELLigence CIM-Plate system (Agilent, Santa Clara, CA, USA). Then, 10% ADS in DMEM/F12 with 0.1% FBS was placed in the lower chamber to stimulate cell migration. Cells (30,000/100 µL) were placed in the upper chamber in DMEM/F12 with 0.1% FBS. Cells migrate through a microporous polyethylene terephthalate (PET) membrane (pore size of 8 μm). Cell migration through PET was recorded by the xCELLigence system (Agilent, Santa Clara, CA, USA). 

### 4.9. Invasion Assay

Cell invasiveness was determined with matrigel-coated transwells (8 µM pore size) (Fisher Scientific, Waltham, MA, USA). RPMI-1640 with 20% FBS and 10% ADS was placed in the lower chamber to stimulate cell invasion. T47D cells (250,000/500 µL) were added to the upper chamber in RPMI-1640 with 0.1% FBS. Cells are invasive if they invade through the membrane coated with matrigel. After 4 days, cells that invaded through the matrigel-based membrane were stained with crystal violet. Cells stained with crystal violet were rinsed several times with tap water. Crystal violet-stained cells were lysed in DMSO for 10 min with rotation. The relative amount of crystal violet was determined by measuring the absorbance of the cell lysate at 560 nm using a BioTek Synergy HTX multi-mode plate reader (BioTek, Winooski, VT, USA).

### 4.10. Statistical Analysis

Two-tailed, paired *t*-tests with confidence intervals of 95% were used to determine statistically significant differences between two groups. Two-way ANOVA, followed by Tukey’s multiple comparisons test, was used to determine statistically significant differences among groups. The results in this study are presented as means ± SE of *n* = 3 number of experiments performed. The data were considered significant if the *p*-value was <0.05.

## 5. Conclusions

To our knowledge, this study showed for the first time that O-ADS induces LAT1 activity and mTOR signaling in ER-positive BC cells. The induction of LAT1 was functionally linked with the induction of mTOR signaling in response to O-ADS. These findings show mechanistic evidence that targeting LAT1 to inhibit mTOR in ER-positive BC could be uniquely effective in obesity. 

## Figures and Tables

**Table 1:** Kinetic parameters of leucine uptake in MCF7 cells in response to L-ADS and O-ADS. Maximal rate of uptake (Vmax) was unchanged. The affinity for leucine was significantly * increased by O-ADS.

Kinetic Parameters	L-ADS	O-ADS
*V_max_* (pmol/mg protein.30 s)	15.55	15.87
*Km* (µM)	163.6 ± 6	82 ± 4 *

## Data Availability

Not applicable.
